# Structure and Novel Functional Mechanism of *Drosophila* SNF in *Sex-Lethal* Splicing

**DOI:** 10.1371/journal.pone.0006890

**Published:** 2009-09-03

**Authors:** Jicheng Hu, Gaofeng Cui, Congmin Li, Cong Liu, Erchang Shang, Luhua Lai, Changwen Jin, Jiwu Wang, Bin Xia

**Affiliations:** 1 Beijing Nuclear Magnetic Resonance Center, Beijing, People's Republic of China; 2 College of Life Sciences, Peking University, Beijing, People's Republic of China; 3 College of Chemistry and Molecular Engineering, Peking University, Beijing, People's Republic of China; 4 Allele Biotechnology & Pharmaceuticals, Inc., San Diego, California, United States of America; Centre de Regulació Genòmica, Spain

## Abstract

Sans-fille (SNF) is the *Drosophila* homologue of mammalian general splicing factors U1A and U2B″, and it is essential in *Drosophila* sex determination. We found that, besides its ability to bind U1 snRNA, SNF can also bind polyuridine RNA tracts flanking the male-specific exon of the master switch gene *Sex-lethal (Sxl)* pre-mRNA specifically, similar to Sex-lethal protein (SXL). The polyuridine RNA binding enables SNF directly inhibit *Sxl* exon 3 splicing, as the dominant negative mutant SNF^1621^ binds U1 snRNA but not polyuridine RNA. Unlike U1A, both RNA recognition motifs (RRMs) of SNF can recognize polyuridine RNA tracts independently, even though SNF and U1A share very high sequence identity and overall structure similarity. As SNF RRM1 tends to self-associate on the opposite side of the RNA binding surface, it is possible for SNF to bridge the formation of super-complexes between two introns flanking *Sxl* exon 3 or between a intron and U1 snRNP, which serves the molecular basis for SNF to directly regulate *Sxl* splicing. Taken together, a new functional model for SNF in *Drosophila* sex determination is proposed. The key of the new model is that SXL and SNF function similarly in promoting *Sxl* male-specific exon skipping with SNF being an auxiliary or backup to SXL, and it is the combined dose of SXL and SNF governs *Drosophila* sex determination.

## Introduction

In *Drosophila melanogaster*, sex determination and differentiation are controlled by the key gene *Sex-lethal* (*Sxl*). *Sxl* is “on” in females (2X; 2A) and “off” in males (X; 2A) [1], [2], and is controlled by the number of X chromosomes [3]. The on/off switch of *Sxl* is regulated at the transcriptional level by four X-linked signal gene products: SISA, SCUTE, RUNT and UNPAIRED, which act through the early *Sxl* promoter *Sxl^Pe^* [4]–[7]. At the cellular blastoderm stage, the *Sxl^Pe^* promoter is shut down, while the late *Sxl* promoter *Sxl^Pm^* is activated in both sexes. In the presence of early *Sxl* protein product (SXL), female-specific splicing of *Sxl* pre-mRNA is maintained in females through autoregulation, and exon 3 of *Sxl* pre-mRNA is removed after splicing [8]. In contrast, since few or no early SXL are present in males, the *Sxl* transcript from *Sxl^Pm^* is spliced by default and results in a non-functional protein.

Genetics studies have shown that the *Drosophila snf* gene is required for female-specific splicing of *Sxl* pre-mRNA in addition to SXL. *snf* functions both maternally and zygotically in regulating *Sxl* pre-mRNA splicing in germline and soma [9], [10]. In germline, females homozygous for the *snf* mutant *snf^1621^* which encodes a protein with an R49H substitution, are sterile and neither the oocyte nor the nurse cells differentiate properly [11], [12]. This female sterility caused by *snf^1621^* can be suppressed by *Sxl^M1^*, a constitutive mutant of *Sxl* which is male-lethal [13]. In contrast, the role of *snf* in sex-determination in the soma can only be inferred by a female-lethal synergistic interaction between *snf* and *Sxl* mutations [9], [10]. The male lethality of *Sxl^M1^* can be partially suppressed by *snf^1621^* in somatic cells, while *snf^1621^* cannot rescue the male-lethal phenotype of *Sxl^M4^* which is characterized by a higher SXL production rate than that of *Sxl^M1^* [13]. In addition, it has been shown that the involvement of *snf* in *Sxl* autoregulation is dose-sensitive, and *snf* becomes rate-limiting for *Sxl* autoregulation when SXL levels are low [14].

The *snf* gene encodes a ∼25 kDa protein (SNF) with two RNA-recognition motifs (RRMs), and SNF was found to be the *Drosophila* homologue of mammalian snRNP components U1A and U2B″ [15]–[17]. Despite extensive investigations of the genetic interactions between *snf* and *Sxl* mutations, the role of *snf* in *Sxl* autoregulation is still poorly understood. In the prevailing model, it is suggested that SNF interacts with SXL as a component of U1 and/or U2 snRNPs, and thus interferes with the normal functions of snRNPs at exon 3 [18], [19]. This model is mainly based on the finding that SXL could form an RNase-sensitive complex with SNF [18]. It has also been proposed that SNF regulates *Sxl* splicing by providing additional interactions between SXL and U1 snRNP, which are critical when SXL levels are low [20]. However, Cline *et al.* suggested that SNF might act as a free protein in regulating *Sxl* splicing since the proposal that SNF functions in *Sxl* regulation only as a part of U1 and/or U2 snRNPs is incompatible with the dose effect of *snf* [14].

In this paper, we report findings that shed light on the role of SNF in *Sxl* autoregulation. We show that SNF can directly prevent pre-mRNA splicing of *Sxl* exon 3 *in vitro*, in the absence of SXL. In addition, we found that SNF possesses a novel dual RNA binding specificity: besides its ability to bind U1 snRNA, SNF can also bind to poly(U) tracts flanking the alternatively spliced *Sxl* exon, as does SXL. The mutant protein (SNF^1621^), encoded by the dominant negative *snf* ^1621^, is unable to bind poly(U) RNA, whereas it binds U1 snRNA the same as the wild type protein. Moreover, we present the solution structures of the two RNA recognition motifs (RRMs) of SNF, and our NMR studies show that RRM1 and RRM2 are involved in poly(U) RNA binding independently and SNF can self-associate through RRM1. Taken together, these results lead to a new model for how SNF regulates *Sxl* pre-mRNA splicing and how it affects sex determination in *Drosophila*, which can explain almost all the previous experimental observations about *snf*.

## Results

### SNF inhibits Sxl exon 3 splicing in vitro

**Figure 1:**
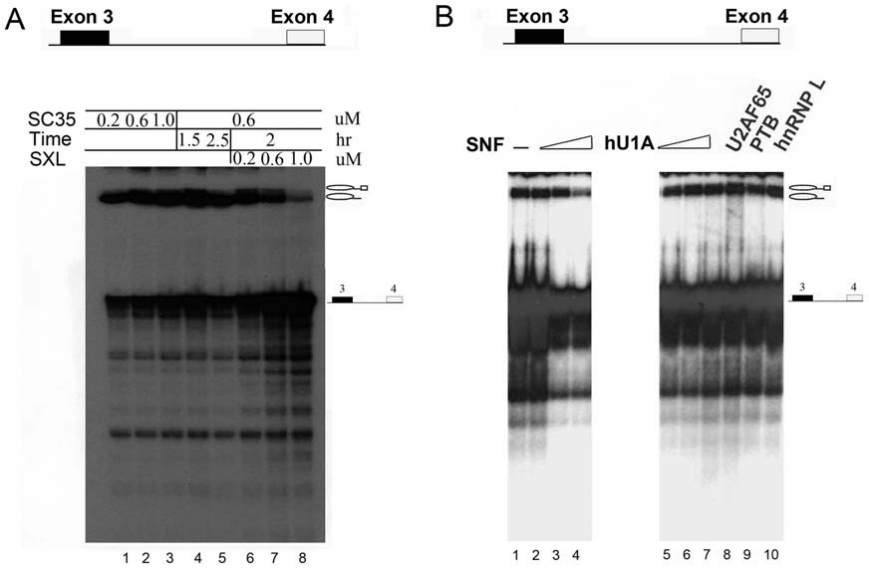
*In vitro* splicing of *Sxl* pre-mRNA. *In vitro* transcribed, radioactively-labeled RNA encompassing exon 3 to exon 4 (E3-4) of *Sxl* pre-mRNA was used as the *in vitro* splicing substrate. The positions of substrate E3-4 and splicing-intermediate RNAs are represented schematically on the right-hand side of the panel. Boxes indicate introns; Lines indicate exons; Loops indicate lariat structures. (A) The effect of the general splicing factor SC35 enhanced efficiency of the splicing reaction (lanes 1–3); and the effect of SXL at increasing concentrations on the splicing of E3-4 (lanes 4–8). (B) Effects of SNF on the splicing of E3-4. Lanes 1 to 4: addition of 0, 3, 9, or 15 µM SNF protein, respectively; lanes 5 to 10, effects of human U1A protein (hU1A) and known polypyrimidine binding proteins U2AF65, PTB, and hnRNP L on the splicing reaction.

To study *Sxl* pre-mRNA splicing, we established an *in vitro Sxl* pre-mRNA splicing assay. We chose the region from exon 3 (including its 3′ splicing site) to the end of exon 4 in *Sxl* pre-mRNA as the splicing substrate (referred to as E3-4), as both the 3′ splicing site and the 5′ splicing site of *Sxl* exon 3 are involved in splicing regulation even though the 5′ splicing site is dominant in regulation [21], [22]. Under standard *in vitro* splicing conditions using Hela cell nuclear extract, the splicing of E3-4 RNA was very inefficient and there was almost no reaction products after two hours (data not shown). Similar attempt has been made previously in establishing *in vitro* splicing assay with *Sxl* transcript from exons 2 to 4, but no splicing could be detected [23]. Fortunately, we found that the *in vitro* splicing of E3-4 was dramatically accelerated by the addition of splicing-enhancer SR proteins (e.g. SC35), as evident by the amounts of splicing intermediates accumulated (Figure 1A, lanes 1–3). However, even with SC35 (lanes 4–5), the second step of *Sxl* splicing was still very slow and the final splicing products were hardly visible (Figure 1A, lanes 4 and 5). Nevertheless, by monitoring the amounts of splicing intermediates, we were able to observe directly the regulatory effect of SXL in *Sxl* splicing. When SXL was added, E3-4 splicing was inhibited in a dose-dependent manner (Figure 1A, lanes 6–8). Thus, the inhibition by SXL should occur at an early splicing step as the amounts of both splicing intermediates were decreased, but the ratio of the two bands remained the same. This result validates our splicing assay as a useful method for studying the regulation of *Sxl* splicing.

To our surprise, we found that the splicing of E3-4 could be inhibited directly by SNF using the established *in vitro* splicing assay, and this inhibition was also dose-dependent, similar to that of SXL (Figure 1B, lanes 2–4). However, the inhibitory effect of SNF was not as strong as that of SXL, and it required 15 times more SNF to achieve a comparable inhibition to that of SXL. As controls, we found that RNA splicing factors U2AF^65^ and hnRNP L did not cause any observable changes in the *in vitro* splicing assay (Figure 1B, lane 8 and lane 10). PTB, which negatively regulates a number of splicing events in human [24], seemed to down-regulate E3-4 splicing slightly (Figure 1B, lane 9). We have also tested human U1A protein, an SNF homologue protein, and no effect of U1A on E3-4 splicing was observed (Figure 1B, lanes 5–7). On the other hand, we also added SNF to splicing reactions with a panel of commonly used *in vitro* splicing substrates, HIV tat for example, and did not observe any significant effect (data not shown). Therefore, *Sxl* exon 3 splicing suppression by SNF seems to be specific.

### SNF directly binds poly(U) while SNF^1621^ does not

**Figure 2:**
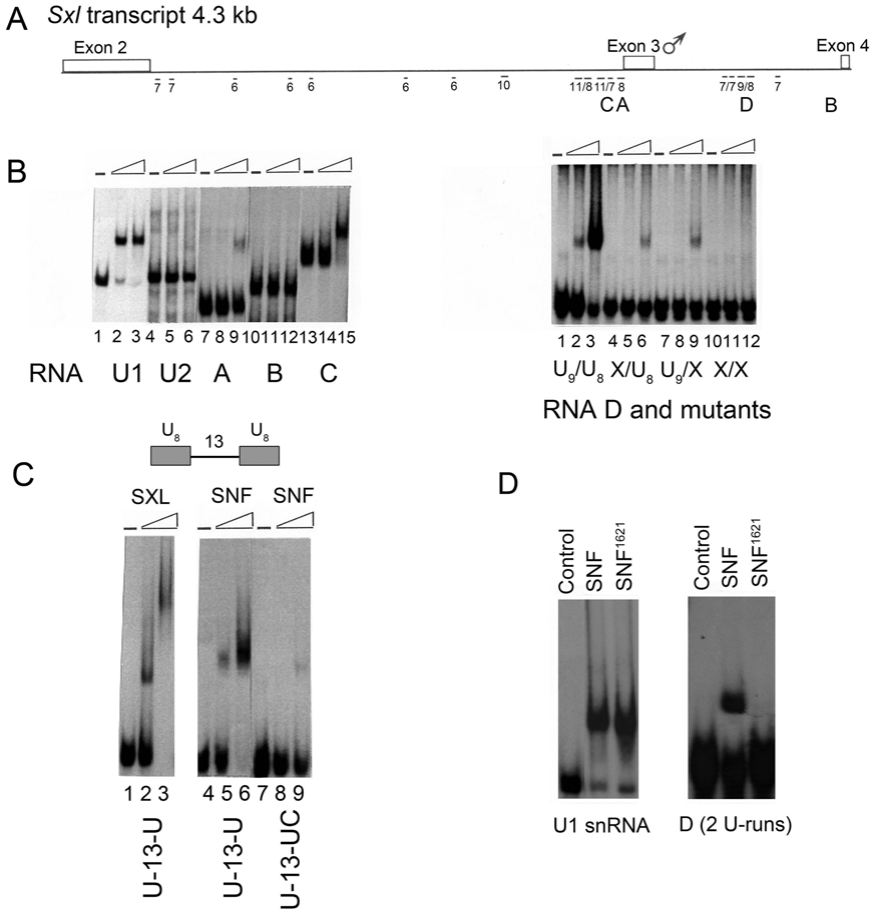
Binding assay for SNF and different RNA fragments. (A) Schematic representation of the exon 2 to exon 4 region of *Sxl* pre-mRNA. All U-runs of at least 6 bases are indicated, and four fragments used as binding substrates are labeled as A, B, C, and D. (B) Band-shift experiments for SNF and U1 snRNA (left, lanes 1–3), U2 snRNA (left, lanes 4–6), RNA A (left, lanes 7–9), RNA B (left, lanes 10–12), RNA C (left, lanes 13–15), RNA D (right, lanes 1–3), and RNA D mutants (right, lanes 4–12). (C) Band shift experiments for SXL and 2U-13 (lanes 1–3), and for SNF and 2U-13 (lanes 4–6), and 1U-13 (lanes 7–9). (D) Band shift experiments for binding of wild-type SNF and mutant SNF^1621^ (R49H) with U1 snRNA and RNA D.

In *Sxl* pre-mRNA, there are multiple polyuridine (poly(U)) sequences scattered in the introns flanking the male-specific exon 3, and four of them contain 8 or more uridine residues (Figure 2A, labeled as RNA A, B, C and D). Previous studies have shown that these poly(U) sequences are critical for SXL to regulate its pre-mRNA splicing, in which SXL inhibits the splicing of exon 3 through a blockage mechanism by binding to poly(U) sequences [21], [25]–[27]. Since SNF can directly regulate *Sxl* pre-RNA splicing in a similar manner to SXL, we decided to explore if SNF can also bind poly(U) sequences.

A band-shift assay was used to test the RNA binding ability of SNF. As expected, SNF could bind U1 snRNA, but did not bind U2 snRNA without U2A′ (Figure 2B) [15], [17]. We then performed an RNA binding experiment in which SNF was mixed with different RNA fragments (RNA A, B, C and D) of the *Sxl* transcript (Figure 2A). Interestingly, we found that SNF binds weakly to RNA A which contains only a single U-run (∼8 poly(U) sequence), and binds strongly to the double U-run-containing RNA C and RNA D, but does not bind RNA B without U-run (Figure 2B). To confirm that such binding is due to the recognition of U-runs by SNF, mutants of RNA D were constructed in which either or both U-runs were changed to UC-runs (Figure 2B). SNF bound mutant RNA D much more weakly when one U-run was changed to a UC-run, and did not bind mutant RNA D when both U-runs were changed to UC-runs (Figure 2B, lanes 1–12, right panel). These results show clearly that the presence of U-runs is necessary for SNF to bind *Sxl* pre-mRNA.

As SNF bound much more strongly to double-U-run-containing RNA than single-U-run-containing RNA, we studied the binding of SNF to different double-U-run-containing RNAs in which the spaces between two U-runs are different. For a consecutive double-U-run-containing RNA, the size of the SNF-RNA complex formed remained unchanged with increasing SNF concentration (data not shown). This is different from SXL which forms a bigger size complex with the same RNA at high concentrations [25], and should indicate that the two consecutive U-runs are simultaneously recognized by one SNF molecule. When the two U-runs were separated by 13 bases (2U-13), the size of SNF-RNA complex formed was still independent of the SNF/RNA ratio (Figure 2C, lanes 4–6), while SXL still formed a bigger complex with the same RNA at high concentrations (Figure 2C, lanes 1–3). Moreover, when the sequence of one of the two U-runs was altered (1U-13), it could still form a complex with SNF and the size was the same as that for 2U-13 (Figure 2C, lanes 7–9). However, the complex of SNF and 1U-13 was less abundant, consistent with a weaker binding affinity. These results support the idea that a strong SNF binding site is composed of two U-run sequences, even if they are separated by more than 10 bases.

To further examine this idea, we separated two U-runs by 120 bases (2U-120) to see if it would still work as a strong binding site (Figure S1, left). Astonishingly, it appeared that a single SNF can still recognize the two U-runs across a significant distance because 2U-120 still formed a single defined complex with SNF, albeit weaker than 2U-13. Furthermore, we compared the binding of SNF to 2U-13-2U and to 2U-13-UC, and found that two complexes could form when there were two double-U-runs (Figure S1, right).

SNF^1621^ has a point mutation in RRM1 (R49H) and functions as a dominant-negative factor in female-specific *Sxl* splicing [19]. We used the same *in vitro* RNA-binding assay to examine the RNA-binding ability of SNF^1621^. With respect to U1 snRNA, SNF^1621^ behaved like the wide type SNF and bound U1 snRNA efficiently (Figure 2D, left, lanes 2–3). However, no complex was observed when SNF^1621^ was mixed with the double U-run-containing RNA D (Figure 2D, right, lanes 3). These results suggest that the poly(U) binding ability of SNF is important for this general splicing component to specifically participate in *Sxl* splicing. As a circumstantial support for this suggestion, human U1A which did not inhibit *Sxl* exon 3 splicing (Figure 1B), bound fly snRNA U1 but not poly(U) RNA (data not shown).

### Solution structures of SNF RRM1 and RRM2

Even though SNF shows a very high amino acid sequence identity to U1A (Figure S4A), SNF is able to recognize both U1 snRNA and poly(U) RNA while U1A is not able to recognize poly(U) RNA. This novel dual RNA recognition ability of SNF led us to study the solution structure of SNF in order to reveal the structural basis for its unique functions.

Near-complete backbone ^1^H/^15^N chemical shift assignments for full-length SNF were obtained (Figure S2). The ^1^H-^15^N correlation peaks of full-length SNF were quite similar to the overlay of those from isolated RRM1 (residues 1–104) and RRM2 (residues 134–216) (data not shown), indicating that each RRM is relatively independent and there lacks inter-domain interaction. Both RRMs of SNF are quite rigid, whereas the linker loop (96–140) is flexible as indicated by low {^1^H}-^15^N NOE values (Figure S3). Although RRM1 and RRM2 have similar molecular weights, the average ^15^N R_2_/R_1_ ratio for RRM1 (∼17.4) and RRM2 (∼9.5) are significantly different, also indicating that RRM1 and RRM2 tumble independently in solution. As the quality of NMR spectra of full-length SNF was very poor, the ^1^H, ^15^N and ^13^C chemical shift assignments for SNF RRM1 and RRM2 were obtained, respectively (BioMagResBank database under accession numbers 6930 and 6844). The structures of SNF RRM1 and RRM2 were also solved separately (Protein Data Bank accession numbers 2K3K and 2AYM). Statistic data indicate that both structures are well defined (Table 1).

**Table 1:** Experimental and structural statistics for the ensembles of 20 structures of SNF RRM1 and RRM2.

Parameters	RRM1	RRM2	
Distance constraints			
Intra-residue (|i−j | = 0)	693		977
Sequential (|i−j| = 1)	315		641
Medium (2 ≤ |i−j| ≤ 4)	157		413
Long-range (|i−j| ≥ 5)	308		701
Ambiguous	1200		950
Total	2673		3682
Dihedral angle constraints			
ϕ	43		32
ψ	45		29
Total	88		61
Hydrogen bond constraints	44		52
Structure statistics (20 structures)			
Violation statistics			
NOE violation (>0.3 Å)	0		0
Maximum NOE violation (Å)	0.26		0.21
Torsion angle violation (>5°)	0		0
Energy			
Mean AMBER energy (kcal mol^−1^)	−5411.0		−4232.9
Mean bond energy	34.3		25.6
Mean angle	149.0		160.3
Mean dihedral	867.8		720.7
Mean VDW	−768.8		−633.9
Ramachandran plot analysis			
Most favored regions	85.9%		84.7%
Additional allowed regions	12.8%		14.3%
Generously allowed regions	0.9%		0.9%
Disallowed regions	0.4%		0.1%
RMSD from mean structure^a^ ^b^			
Backbone atoms (Å)	0.64±0.16^c^		0.49±0.18
All heavy atoms (Å)	1.12±0.15^c^		1.08±0.17
Regular secondary structures (Å)^a^ ^b^			
Backbone atoms (Å)	0.37±0.11^c^		0.21±0.04
All heavy atoms (Å)	0.92±0.16^c^		1.02±0.20

a The average RMSD between the 20 structures of the lowest AMBER energies and the mean coordinates (±standard deviation).

b Calculated with PROCHECK_NMR [61].

c Residues 1–83 in SNF RRM1 were used in the calculation.

**Figure 3:**
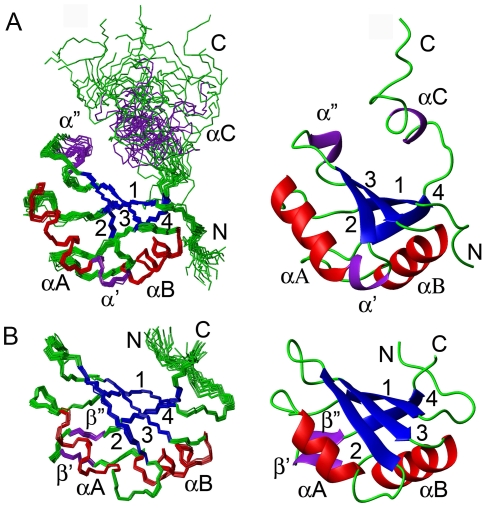
Solution structures of SNF RRM1 and RRM2. The structures of SNF RRM1 and RRM2 are shown in (A) and (B), respectively. Backbone traces of 20 superimposed conformers with lowest SNF RRM1 and RRM2 AMBER energies are shown on the left; Ribbon representations of the energy minimized mean structure of SNF RRM1 and RRM2 are shown on the right. The secondary structures are labeled. Additional α-helices and β-strands are shown in purple.

The structures of SNF RRM1 and RRM2 are characteristic of typical RNP-type RBDs [28] comprising a four-stranded anti-parallel β-sheet packed against two α–helices (Figure 3). Two additional α–helices (α′ and α″) are inserted in the loop regions of RRM1. Helix α′ directly follows αA with a kink at Ile^30^, and helix α″ is inserted in loop3 (Figure 3). Residues 88–95 in RRM1 form an α-helix (αC) which is flexible, as indicated by the fact that {^1^H}-^15^N NOE values for residues in helix αC are significantly lower than those in the core regions of RRM1 (Figure S3). The other secondary structure elements (1–83) are well defined with root mean square deviation (RMSD) values for backbone heavy atoms of 0.4 Å. In RRM1, the conserved RNP-1 and RNP-2 submotifs lying in the center of strands β1 and β3 contain two conserved aromatic residues (Tyr^10^ and Phe^53^) [29]. Compared to RRM1, RRM2 lacks helices α′, α″ and αC, whereas two short β–strands (β′ and β″) inserted in the loop connecting αB and β4 form a small anti-parallel β-sheet (Figure 3).

SNF RRM1 has a close resemblance to U1A RRM1 (Figure S4B), and their backbone C^α^ atoms align well with a 1.6 Å RMSD (residues 7–86 of SNF, and residues 10–90 of U1A). Calculation of electrostatic potential surfaces showed that RRM1s of both U1A and SNF are highly charged with large clusters of positive charges on the RNA binding surface (Figure S4B). In general, SNF possesses a similar charge distribution on the surface of RRM1 to that of U1A. A small difference is that the residue Arg^83^ in U1A, which contacts U1 snRNA directly, is replaced with a Gln in SNF (Figure S4B). Another notable difference is that SNF αC points away from the β-sheet surface and does not make interactions with residues in the β-sheet surface, while U1A αC forms a small hydrophobic core with residues in the β-sheet surface (close conformation) and it swings away from the β-sheet surface by ∼130° upon U1 snRNA binding [29].

The RMSD of C^α^ atoms between SNF RRM2 (residues 141–216) and U1A RRM2 (residues 207–282) is 1.5 Å. SNF RRM2 lacks the N-terminal capping box in helix αA, which is an important structural motif in U1A RRM2 and other RRM proteins [30]–[32]. On the exposed β-sheet surface, U1A RRM2 has more negatively charged residues than SNF RRM2 does (Figure S4C).

### Binding of SNF to U1 snRNA and Poly(U) RNA

**Figure 4:**
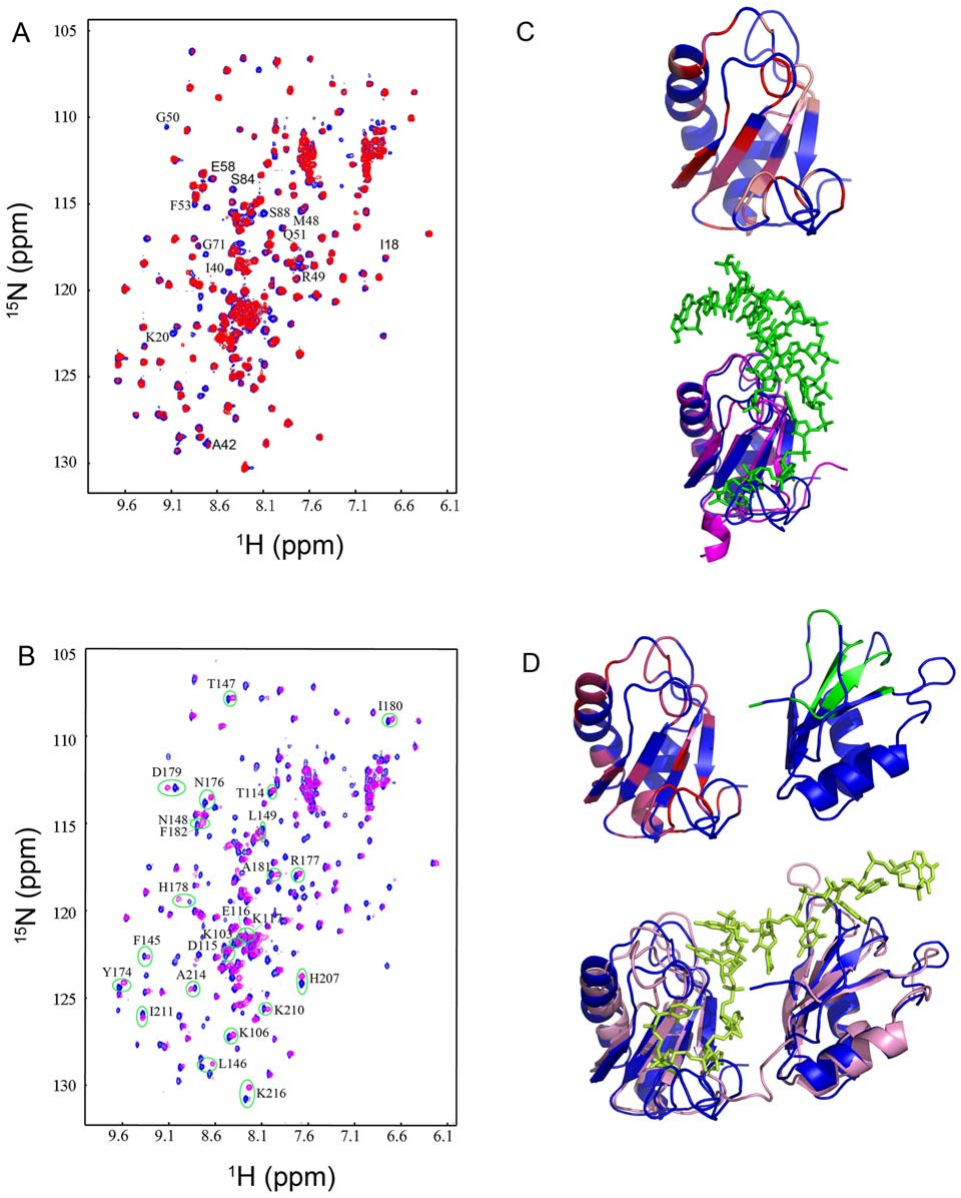
Binding of SNF to U1 snRNA and poly(U) RNA. Overlay of the 2D ^1^H–^15^N HSQC spectra of free SNF with that of SNF/U1hpII RNA (A) and SNF/2U-run RNA (B). In (A), residues displaying large peak intensity changes (less than 33% of free protein) are labeled. In (B), residues exhibiting obvious ^1^H-^15^N chemical shift changes (Δδ_comb_>0.04 ppm; Δδ_comb_  =  (Δδ_HN_^2^ + (Δδ_N_/6.5)^2^)^1/2^) in SNF RRM2 are labeled. (C) Mapping of U1hpII RNA binding surface on SNF. The perturbed residues are mapped on the structure of SNF RRM1 (I_free_/I_U1_ ≥3.0 are shown as red, 2.2 < I_free_/I_U1_ < 3.0 are shown as pink). The structures of SNF RRM1 (blue) and U1A (yellow, with U1hpII RNA) are aligned for comparison. The bound U1hpII RNA in the U1A/U1hpII RNA complex is shown in green. (D) Mapping of the 2U-run RNA binding surface on SNF. Residues in SNF RRM1 that display significant signal broadening (I_free_/I_U8_ ≥5.0 are shown as red, 2.5 < I_free_/I_U8_ < 5.0 are shown as pink) and in RRM2 that display obvious chemical shift changes (Δδ_comb_>0.04 ppm, green) are shown. The structures of SNF RRM1 and RRM2 (blue) are aligned with the structures of RRM1 and RRM2 in the SXL/GUUGUUUUUUUU complex (violet). The bound RNA is shown in lemon.

The interactions of full-length SNF with U1 snRNA and poly(U) RNA were analyzed by NMR chemical shift perturbation experiments, in which 2D ^1^H-^15^N HSQC spectra of H^2^/^15^N/^13^C-labeled SNF were recorded with stepwise titration of RNAs (Figure 4). With the addition of the U1 snRNA stem-loop II segment (U1hpII), we observed significant exchange broadening of NH signals without chemical shift change (Figure 4A). These residues are all located in SNF RRM1 and the linker loop (Figure S5A). Locations of these residues on the structure of SNF RRM1 define the binding surface of SNF to U1 snRNA, and it is clear that this binding surface is similar to that of U1A to U1 snRNA (Figure 4C) [29], [33]. In contrast, no NH signal of residues in SNF RRM2 displays significant change in chemical shift or peak intensity during U1hpII titration (Figure 4A). These results demonstrate that SNF RRM1 is necessary and sufficient for binding U1hpII.

Upon the addition of a double-U-run containing RNA (2U-run, sequence 5′UUUUUUUUAUUUUUUUU3′), the NH chemical shift change pattern indicates that both RRMs of SNF are involved in poly(U) binding, unlike U1 snRNA binding (Figure 4B, 4D). In RRM1, many NH cross-peaks display significant intermediate exchange line-broadening (Figure 4B). These residues are mainly located in the β-sheet surface, the edge of α–helices and loop regions. While in RRM2, a number of NH cross-peaks shifted gradually as the RNA concentration increased (Figure 4B). These residue are mainly located in the vicinity of β1, β3, and the C-terminus of RRM2, along with the linker loop between RRM1 and RRM2 (Figure 4B, 4D, S5B, S5C). The poly(U) RNA binding surfaces derived from NMR titration data for SNF RRM1 and RRM2 are roughly similar to those of SXL RRM1 and RRM2 revealed by X-ray crystallography (Figure 4D) [34]. In addition, these observations suggest that RRM1 and RRM2 should bind U-runs independently, and it is probable that each RRM binds a different U-run. We have also titrated RRM2 alone with 2U-run RNA and observed a similar chemical shift perturbation pattern to that of RRM2 in the full-length protein (data not shown). This further proves that the two RRMs of SNF bind poly(U) RNA independently.

The binding of SNF to U1hpII and poly(U) RNA RNA (2U-run) was also probed by the SPR measurements (Figure S6). The results clearly show that the interaction between SNF RRM1 and U1hpII involves a high association rate and low dissociation rate (Figure S6A). The apparent equilibrium dissociation constant *K_d_* value extracted from the SPR data was ∼4.4 nM, which is about 2 orders higher than the *K_d_* for U1A RRM1 binding U1hpII [35], [36]. Meanwhile, the *K_d_* for SNF binding 2U-run RNA is ∼0.3 µM, which is about 2 orders higher than the reported *K_d_* for SXL binding 1U RNA (5′-GUUUUUUUUC- 3′) [37]. Scatchard-plot analysis using results from the sensorgrams also confirmed a 1∶1 complex between SNF and 2U-run RNA. As expected, SNF RRM1 alone binds 2U-run RNA with a *K_d_* of ∼6 µM, about 20-fold weaker in binding affinity. The binding affinity of SNF RRM2 to 2U-run RNA is estimated to be at low mM range from NMR titration data, which is much weaker than that of SNF RRM1.

### Self-association interface of SNF RRM1

The 2D ^1^H–^15^N HSQC spectrum of SNF at 0.4 mM revealed that NH peak intensities of residues in the RRM1 were significantly lower than those of residues in RRM2 (Figure S2). When the concentration of SNF was raised from 0.4 mM to 2 mM, about half of the NH signals in the 2D ^1^H-^15^N HSQC spectrum, mainly from residues in RRM1, disappeared (data not shown). These results suggest that SNF self-associates through RRM1, and this is consistent with the above mentioned observation that the average ^15^N R_2_/R_1_ ratio of RRM1 is much bigger than that of RRM2 even though the two RRMs are about the same size. In addition, analytical ultracentrifugation analysis indicates that SNF RRM1 could self-associate into dimeric and higher oligomeric species (Figure S7). The apparent equilibrium dissociation constant *K_d_* for monomer-dimer equilibrium of SNF RRM1 is estimated to be a few hundred micromolar.

**Figure 5:**
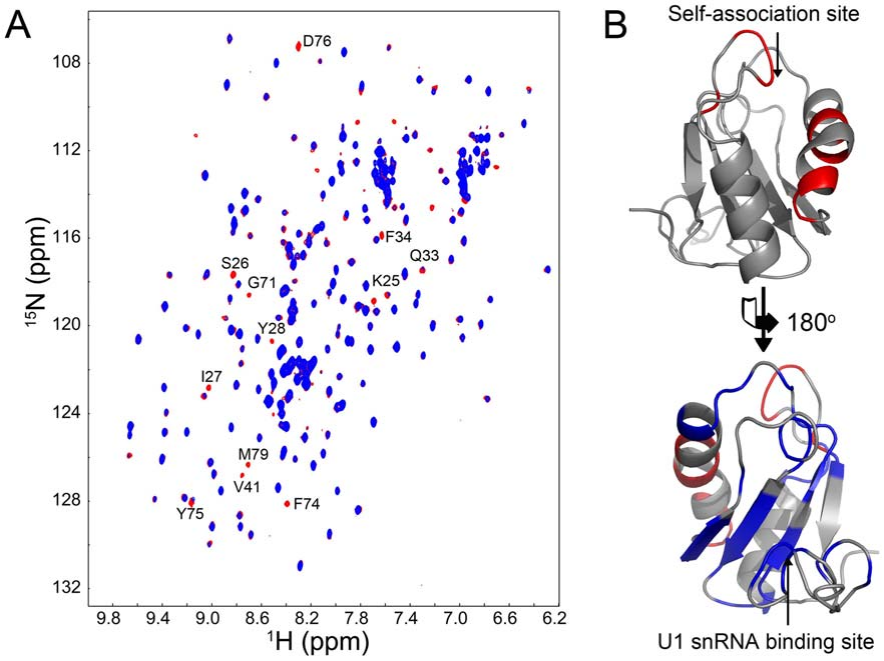
Self-association interface of SNF RRM1. (A) The superimposed 2D ^1^H–^15^N HSQC spectra of SNF recorded at 0.39 mM (blue) and 0.035 mM (red). Residues displaying significant NH signal intensity changes are labeled. (B) Residues displaying significant NH signal intensity changes are mapped onto the structure of RRM1 (red, upper side); U1 snRNA binding site is also shown (blue, lower side) indicating that the RNA binding site and self-association site do not interfere with each other. The two structures diverse 180°.

Overlay of 2D ^1^H–^15^N HSQC spectra of SNF at different concentrations (0.035–0.39 mM) revealed that some NH cross-peaks (e.g. residues Lys^25^, Ser^26^, Leu^27^, Tyr^28^, Gln^33^, Phe^34^, Gly^35^, Phe^74^, Tyr^75^, Asp^76^ and Met^79^) displayed significant concentration-dependent line broadening (Figure 5A). These residues could be mapped to one area of SNF RRM1, which composes the self-association surface. Interestingly, the self-association surface does not interfere with U1 snRNA and poly(U) RNA binding surfaces of SNF RRM1 (Figure 5B).

## Discussion

Among a number of genes that could influence *Sxl* function at the post-transcription level, *snf* is perhaps the most specific and well studied. However, the underlining mechanism by which male-specific exon (exon 3) is efficiently skipped in female regulated by SNF is still not well understood. The current prevailing model suggests that SNF acts as a component of U1 snRNP and provides interaction between U1 snRNP and SXL (which is bound to the RNA surrounding the male exon (exon 3)); this interaction leads to the formation of an abortive pre-splicing complex for exon 3 and the alternative exon 2–4 female-specific splicing proceeds by default [20]. However, this model does not explain why the SNF^1621^ mutant does not affect the interaction between U1 snRNP and SXL [20], while it abolishes male-specific exon skipping for *Sxl* pre-mRNA splicing in female flies but has no effect in male flies [11], [12]. In addition, this model is mainly based on the observation that SXL and SNF can form an RNA-sensitive complex [18]. However, the observation that RNAase digestion disrupts the complex indicates that SNF lacks direct interaction with SXL and that the complex is possibly formed through bridge of RNAs [18]. Lack of direct interaction between SNF and SXL was also revealed by our NMR chemical shift perturbation experiment, which can detect extremely weak protein-protein interactions (*K*_d_>10^−4^ M) [38]. Our studies showed that titrating SXL into SNF sample resulted in no change to the 2D ^1^H-^15^N HSQC spectrum of SNF, and vice versa (data not shown). Thus, as SNF does not interact with SXL directly, it is questionable whether SNF could provide additional interactions between SXL and U1 snRNP [20]. Moreover, Cline *et al.* found that the dose effect of *snf* is incompatible with a role for SNF participating in *Sxl* splicing autoregulation only as an integrated component of U1 snRNP or U2 snRNP [14]. They proposed that that SNF may participate in regulating *Sxl* splicing as a free protein [14], but this cannot be reconciled with the prevailing model. Consistent with this idea, it was later reported that non-snRNP associated SNF can be detected in *Drosophila*, suggesting that SNF is able to involve in other interactions independent of U1 or U2 snRNP [39].

In this work, using *Sxl* E3-4 pre-RNA as the substrate of an *in vitro* splicing assay, we have shown that SNF can directly inhibit the generation of exon 3 splicing intermediates of *Sxl* pre-mRNA *in vitro*, and that this inhibition by SNF is dose-dependent (Figure 1B). This observation is very similar to that for SXL, although the inhibitory effect of SNF is less efficient than that of SXL. These data lend support to the idea that SNF regulates *Sxl* at the step of splicing and that SNF can do so without SXL, in good agreement with the gene-dose-effect result reported by Cline *et al.* [14].

**Figure 6:**
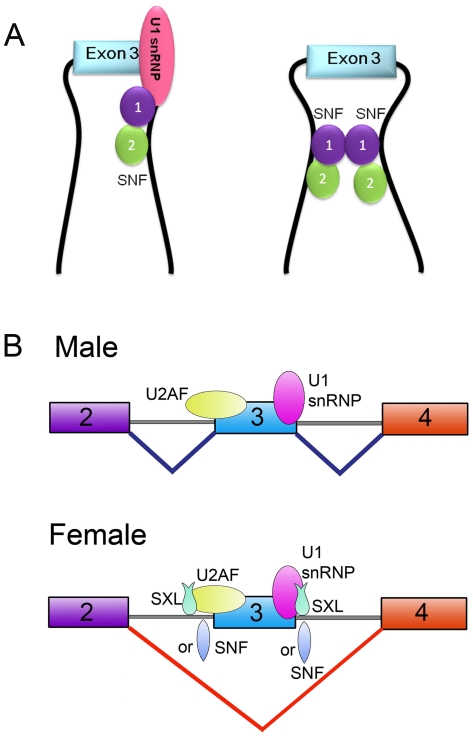
A new model for the role of SNF in *Sxl* autoregulation. (A) Models illustrating how SNF specifically regulates *Sxl* splicing. The pre-mRNA is represented as a thick black line. SNF RRM1 and RRM2 are shown in the purple circle and green circle, respectively. (B) Proposed model for the role of SNF in *Sxl* autoregulation. SNF binds poly(U) tracts in *Sxl* pre-mRNA and acts as an auxiliary or backup for SXL, and provides “dose compensation” for SXL when SXL protein levels are low.

A combination of data from *in vitro* biochemical analysis and NMR studies is presented here to reveal the function mechanism of SNF in regulating *Sxl* splicing. Our studies show that besides its ability to bind U1 snRNA, SNF can also bind to poly(U) RNA tracts flanking exon 3 in *Sxl* pre-mRNA (Figure 2). Binding of U1 snRNA is only through RRM1, whereas both SNF RRM1 and RRM2 are independently utilized to bind poly(U) RNA (Figure 4). Moreover, we have showed that SNF RRM1 and RRM2 tumble independently in solution and that SNF can self-associate via RRM1 through a surface on the side opposite the RNA binding surface (Figure 5). Based on these results, we predict that SNF is capable of forming two kinds of super-complex. First, one SNF molecule could binds poly(U) RNA tracts flanking exon 3 while it also binds another SNF molecule in U1 snRNP to form a super-complex via RRM1-RRM1 interaction (Figure 6A). As revealed by the EM model structure of U1 snRNP, the self-association surface of SNF is exposed and is not blocked by other components in U1 snRNP [40], thus the formation of this super complex is possible. Secondly, as both introns flanking exon 3 of *Sxl* pre-mRNA have multiple poly(U) RNA tracts, it is also possible for two (or more) SNF molecules that bind at different sides of exon 3 to associate through their RRM1 domains (Figure 6A).

The first super-complex is similar to that proposed for SXL which binds poly(U) RNA and U1 snRNP simultaneously to inhibit the splicing of exon 3 [20]. Thus, it is possible that SNF possesses the ability to promote exon 3 skipping in a way similar to SXL. This is consistent with *in vitro* splicing results, which shows that SNF can directly inhibit exon 3 splicing in a similar fashion to SXL. The second super-complex is similar to the looping-out model proposed for PTB to repress splicing of the *c-src* neuron-specific N1 exon, in which PTB can multimerize and bring introns flanking the N1 exon together [41], [42]. Interestingly, we found that PTB protein has a small but detectable inhibitory effect in our *in vitro Sxl* splicing assay (Figure 1).

Taken together, a new model for the role of SNF in *Sxl* autoregulation can be readily proposed based on our results along with previous study results: It has been demonstrated that SNF functions only when the “master switch” protein SXL does not have a strong presence and the whole auto-regulation system needs a “jump-start” [43]. In addition, SNF shows a rate-limiting effect on *Sxl* splicing regulation when SXL levels are low [14]. Thus, SNF should be required in the early period after the late *Sxl* promoter *Sxl* ^Pm^ activation in female fly. At this time, only low level of early SXL protein is present, which by itself is insufficient for the activation or maintenance the female-type *Sxl* splicing [9], [44]. Meanwhile, the cells have large quantity of maternal and zygotic SNF [9], [10]. It is likely that there is not enough SXL to occupy all the poly(U) tracts necessary for inhibiting exon 3 splicing, and SNF can bind those unoccupied poly(U) tracts without competing with SXL. As SNF can also directly inhibit exon 3 splicing in a similar fashion to SXL, it can compensate the dose shortage of early SXL. Therefore, it is the combined dose of SXL and SNF regulates the female-specific *Sxl* transcript splicing in the early period (Figure 6B). As more late SXL protein is produced to a level that SXL can maintain female-type splicing by itself, the effect of SNF in *Sxl* splicing regulating will be overshadowed by SXL and thus no effect could be observed for SNF [43]. This is because that SNF has to compete with large amount of SXL for the poly(U) tracts at this stage, while the binding affinity of SNF is much weaker than that of SXL.

Different from the old model, our new model is consistent with the proposal that SNF participates in regulating *Sxl* splicing as a free protein instead of a component of U1 snRNP [14]. It provides a reasonable explanation for the dose effect observed for SNF in regulating *Sxl* splicing [14]. In addition, a direct interaction between SNF and SXL is no longer necessary in this new model. As both SNF and SXL can bind poly(U) RNA tracts flanking the *Sxl* exon 3, it is very likely that the previously described SNF/SXL complex is formed by the bridge of poly(U)-containing RNA without direct interaction between SNF and SXL [18]. This interpretation is also in agreement with the observation that *Sxl* transcripts exist in SNF/SXL complex [18]. Moreover, the beauty of our new model lies that it provides reasonably explanations to almost all the previous results about SNF at molecular level.

Most of the important evidences for defining the function of *snf* in regulating *Sxl* splicing came from studies of mutant *snf^1621^* which causes female sterility and displays a dominant negative effect [19]. According to our model, the female-specific splicing is controlled by the combined dose of SNF and SXL. As SNF^1621^ cannot bind poly(U) RNA tracts, it cannot inhibit the splicing of exon 3 and thus it cannot compensate the dose shortage of early SXL. As a result, male-specific splicing proceeds by default and non-functional male-type SXL protein is produced in female, which in turn causes female sterility. In addition, as SNF^1621^ has a single residue substitution at R49 (R49H) and this residue is not located on the self-association surface, it is expected that the mutation R49H should not affect the self-association even though it abolishes the poly(U) RNA binding ability of SNF. A molecule of SNF^1621^ can still associate with a wild-type SNF molecule in competing with other wild-type SNF molecules, which results in hybrid super-complex that is not functional. Therefore, SNF^1621^ can have a negatively effect on the function of wild-type SNF, which explains why *snf^1621^* displays a stronger dominant lethal-synergistic interaction with *Sxl* than the null allele *snf^J210^* and acts as a gain-of-function mutant [19].

Furthermore, it was reported that *snf^1621^* could suppress the male lethality associated with the constitutive mutant *Sxl^M1^*, while it could not rescue another constitutive mutant *Sxl^M4^* [13], [45]. Both *Sxl^M1^* and *Sxl^M4^* mutants cause production of female-type SXL in male flies at the early stage, and the major difference between them is that *Sxl^M1^* has a lower female-type SXL production rate than *Sxl^M4^* [45]. It is possible that the amount of female-type SXL produced in male flies is insufficient for *Sxl^M1^* while it is sufficient for *Sxl^M4^*, which means SNF is required for compensating the dose shortage of SXL in *Sxl^M1^* but not in *Sxl^M4^*. Thus, the negative effect of *snf^1621^* is displayed in *Sxl^M1^* mutant and *snf^1621^* can rescue the male lethality of *Sxl^M1^* but not *Sxl^M4^*.

In conclusion, our study results reveal a novel role for SNF in *Sxl* autoregulation: in addition to its role in snRNPs, SNF binds directly to poly(U) RNA in introns flanking exon 3 in *Sxl* pre-mRNA and directly regulates *Sxl* splicing, similar to SXL. To our knowledge no other RNA-binding protein has been reported to have similar dual binding capabilities as SNF. SXL and SNF bind to the same poly(U) RNA sequence with such subtle differences that determine their functions in *Sxl* splicing being either dominant or auxiliary/backup. The structure, self-association, and novel dual RNA-binding specificity of SNF reported here, not only form a foundation for understanding its role in *Sxl* autoregulation, but also establish a mechanistic framework that will attract additional studies to delineate the process in the future.

## Materials and Methods

### DNA constructs, RNA and mutants

Plasmids for constructing GST-hnRNP L, GST-PTB, GST-U2AF65 and GST-U1A were gifts from the laboratories of G. Dreyfuss (University of Pennsylvania), M. Garcia-Blanco (Duke University Medical Center), M. Green (University of Massachusetts Medical Center) and S. Mount (University of Maryland), respectively. Generation of GST-SNF, GST-SXL, and SNF^1621^ constructs has been described previously [46]. Small *Sxl* RNAs, RNA A, B, C and D, (containing *Sxl* sequences from the restriction sites *Pvu*II (9278) to *Afl*II (9373), *Eco*RV (10355) to *Bsp*MII (10448), *Sty*I (8255) to *Pst*I (10139), and *Spe*I (9809) to *Afl*II (9892), respectively) were cloned into plasmid pGEM2, and transcribed under the control of the SP6 promoter. Constructs encoding RNA D mutants, U-13-U, and U-120-U were created by ligating annealed DNA oligos into pGEM4 (Promega). To make the SNF, SNF RRM1, and SNF RRM2 constructs, the *snf* gene (Met^1^-Lys^216^) and *snf* RRM1 (Met^1^-Lys^104^) were cloned into a pET-21d(+) expression vector, and *snf* RRM2 (Ala^134^-Lys^216^) was cloned into a pET-28a(+) expression vector.

### In vitro splicing assays

*In vitro* splicing reactions were performed as described by Valcárcel *et al.* [47], [48]. Purified RNA samples were resolved on PAGE containing 6 M urea as previously described [49].

### In vitro RNA binding assays

RNAs were generated by *in vitro* transcription using T7 or Sp6 RNA polymerase (Promega) with P^32^-ATP or P^32^-GTP. Labeled RNAs were precipitated before mixing with the desired amount of protein in binding buffer and run on 4% native polyacrylamide gels as described previously [25]. Binding assays were performed using a high protein/RNA ratio, and protein concentrations were in the order of 1 µM.

### NMR spectroscopy and assignments

The NMR samples contained about 0.4 mM ^15^N- or ^15^N/^13^C- or ^2^H/^15^N/^13^C labeled SNF (RRM1 and RRM2). The buffer contained 1 mM EDTA, 0.01% NaN_3_, 0.006% 2,2-dimethyl-2-silapentane-5-sulfonate (DSS), 50 mM PBS, 90% H_2_O, and 10% D_2_O, at pH 7.2. All NMR data were collected at 298 K on a Bruker AVANCE 600 spectrometer. Backbone sequential and side-chain assignments were obtained using standard 3D NMR experiments [50]. Proton chemical shifts were referenced using DSS, whereas ^13^C and ^15^N chemical shifts were referenced indirectly to DSS [51]. NMR spectra were processed using NMRPipe [52] and analyzed using NMRView [53].

### Structure calculation

NOE constraints were obtained from automated analysis of NOESY spectra using the computer program SANE [54]. Angle constraints (φ and ψ) of the secondary structure were derived using TALOS [55]. Hydrogen bonds were assigned based on analysis of NOEs and secondary structure predictions by CSI [56] and TALOS [55].

The initial structures were calculated with the CANDID [57] program by using only NOE distance constraints. Hydrogen bond constraints and dihedral angle constraints were then added for CYANA [58] calculations. After several rounds, 200 structures were calculated and 100 structures with lowest target function were selected for further refinement using the AMBER program (version 7.0) [59]. The 20 structures with the lowest AMBER energy were used for the final analysis. The final structures were analyzed by using MOLMOL [60] and assessed by using PROCHECK-NMR [61].

### RNA binding

Two RNA sequences 5′CUUGGCCAUUGCACCUCGGCUGAGT3′ (U1hpII) and 5′UUUUUUUUAUUUUUUUUU3′ (2U-run) were synthesized and purified by Allele Biotechnology & Pharmaceuticals Inc. RNAsin was added to prevent RNA degradation. For U1hpII binding, a series of 2D ^1^H-^15^N HSQC experiments were carried out by adding this RNA oligonucleotide to the SNF sample to reach a final protein:RNA ratio of 1∶7. For 2U-run RNA binding, the final ratio was 1∶15.

### Other experimetal methods

Experimetal methods about NMR relaxation measurements, Biosensor analysis, and analytical ultracentrifugation can be found in supplemental file S1.

## Supporting Information

Supplemental File S1 supplemental file
(0.04 MB DOC)

Figure S1 RNA binding assay for SNF and U-120-U (with two U-runs separated by 120 bases), U-120-UC (with one U-run), 2U-13-2U (with two double-U-runs separated by 13 bases) and 2U-13-UC (with one double-U-run) RNA substrates.
(0.63 MB TIF)

Figure S2 The 2D 1H-15N HSQC spectrum of full-length SNF at pH 7.2. Assignments are labeled.
(1.41 MB TIF)

Figure S3 Backbone dynamics of full-length SNF. R1, R2, and heteronuclear {1H}-15N NOE values are plotted against residue numbers.
(0.28 MB TIF)

Figure S4 Sequence alignment and surface charge comparison. (A) Structure based sequence alignment of RRM1 and RRM2 in SNF (residues 7–86 and residues 142–216), U1A (residues 10–90 and residues 207–282) and SXL (residues 125–203 and residues 211–291). Conserved residues are shown in red. The secondary structure is displayed at the top. Residues involved in forming RNP1 and RNP2 are highlighted in green. (B) Comparison of the surface charge of SNF RRM1, U1A RRM1 and SXL RRM1. (C) Comparison of the surface charge of SNF RRM2, U1A RRM2 and SXL RRM2. The alignment of the three structures is shown on the left (SNF: grey; U1A: khaki; SXL: pink). Surface charge distribution of SNF RRM1, U1A RRM1 and SXL RRM1 are shown from left to right. (D) Surface charge distribution of U1A/U1hpII RNA complex (left) and SXL/GUUGUUUUUUUU complex (RRM1 shown in the middle, RRM2 shown on the right). Negatively charged residues are shown in red, and positively charged residues are shown in blue.
(3.87 MB TIF)

Figure S5 Bar plots displaying SNF residues that change in peak intensity or chemical shift on titration with U1hpII RNA,poly(U) RNA and self-association. (A) NH signal intensity ratio between free SNF and SNF/U1hpII (1∶7). The residues that display significant concentration-dependent NH peak intensity changes include I18, K20, D39, I40, A42, M48, R49, G50, Q51, F53 E58, M79, S84, S88 and K93 (Ifree/IU1>3). (B) NH signal intensity ratio between free SNF and SNF/poly(U) RNA (1∶15). The cut off is set to 10. The residues that display significant concentration-dependent NH peak intensity changes include M3, Y10, N15, K19, K25, I37, Y83, I90, V91, A92, K93, F98, V105, K109, D115, K121 and K122 (Ifree/IU8>5). (C) Changes in average NH chemical shifts of SNF RRM2 (plus the linker loop) on titration with poly(U) RNA (1∶15). The following residues exhibit obvious NH chemical shift changes (Δδcomb>0.04 ppm): K103, K106, T114, D115, E116, K117, F145, L146, T147, N148, K149, V174, N176-F182, H207, K210, I211, A214 and K216. (D) NH signal intensity ratio of SNF at 0.035 mM and 0.39 mM. The residues that display significant concentration-dependent NH peak intensity changes include K25, S26, L27, Y28, Q33, F34, G35, F74, Y75, D76 and M79 (I0.035 mM/I0.39 mM>3).
(0.61 MB TIF)

Figure S6 SPR analysis of the interactions between SNF (or SNF RRM1) with U1hpII RNA and poly(U) RNA. SPR analysis was carried out using BIACORE 3000 (Biacore), as described in Materials and Methods. Interactions between U1hpII RNA and SNF RRM1, poly(U) RNA and SNF, poly(U) RNA and SNF RRM1 are shown in A, B and C, respectively. Five different concentrations of protein injected over the RNA surfaces are shown in the right side. Scatchard-plot analysis of the protein-RNA interactions were carried using results from the above sensorgrams, and it is found that the number of binding sites (n) on poly(U) RNA is 1.06 for SNF and 1.20 for SNF RRM1, which are all close to 1∶1 binding stoichiometry.
(0.82 MB TIF)

Figure S7 Analytical ultracentrifugation analysis of SNF RRM1 (A) and SNF (B). The protein concentration was about 0.1 mM for SNF RRM1 and 0.09 mM for SNF (2H, 15N, and 13C triple labeled sample), respectively.
(0.32 MB TIF)
