# Supplementary material for: Structure and Novel Functional Mechanism of Drosophila SNF in Sex-Lethal Splicing
Source: PLoS One. 2009 Sep 3;4(9):e6890. doi: 10.1371/journal.pone.0006890 (PMC2731243; doi:10.1371/journal.pone.0006890)
Supplement: Supplemental File S1 — supplemental file (0.04 MB DOC) [file pone.0006890.s001.doc]

**Supporting information**

**Supporting Materials and Methods**

***NMR relaxation measurements***

The longitudinal relaxation rates (*R*_1_), transverse relaxation rates (*R*_2_), and steady-state heteronuclear {^1^H}–^15^N NOE values of SNF were determined. The experiments were performed on a Bruker AVANCE 600 MHz NMR spectrometer at 25 °C using ^15^N/^2^H double-labeled samples. For the *R*_1_ and *R*_2_ measurements, 512 (^1^H) and 100 (^15^N) complex data points were collected with 16 transients/increment and a recycle delay of 2.5 s. The delays used for the *R*_1_ experiments were 10 (×2), 100, 250, 400, 500, 650, 800, 1000, 1500 and 2000 ms, and those used for the *R*_2_ experiments were 4.162 (×2), 20.81, 41.62, 62.42, 70.75, 81.23, 104.04, 145.66, 166.46 208.08 and 291.31 ms. The {^1^H}–^15^N NOE experiments with 64 transients for data collection were performed in the presence and absence of a 3 s proton presaturation period prior to the ^15^N excitation pulse and using recycle delays of 2 and 5 s, respectively.

***Biosensor analysis***

Binding experiments were carried out using BIACORE 3000 (Biacore, Inc., Piscataway, N.J.) at 25°C. The running buffer (10 mM HEPES [pH 7.4], 150 mM NaCl, 3 mM EDTA, 0.05% surfactant P-20 for U1hpII; 50 mM PBS [pH 7.0], 1 mM EDTA, 0.05% surfactant P-20 for poly(U) RNA) was filtered and degassed every time before use. SA sensor chips (Beijing Hrbio Biotechnology Co., Ltd) was used to immobilize RNAs (5’-GGCUUGGCCAUUGCACCUCGGCUGAGUU-3’ and 5’-CCCCUCCCCUUUUUUUUUAUUUUUUUU-3’) carrying a 5’-biotin tag (Shanghai GenePharma Co., Ltd). U1 RNA was diluted to a final concentration of 20 μM in DEPC water followed by heating at 80 °C for 10 min and cooling to room temperature to allow annealing of the stem. Both RNAs were immobilized an intermediate density RNA surface (120–150 resonance units, RU) which would yield sufficient signal. One flow cell was kept as a control surface. The recombinant protein samples were diluted in running buffer to the concentrations described in each sensorgram and injected at a flow rate of 50 μl/min for 1 min. The RNA-protein complex was disrupted by using a 1-min injection of 2 M NaCl at 20 μl/min.

SPR signals at steady state were used for Scatchard plot analysis using equation: RU/C = m**K_a_* –RU**K_a_*, where m = n*RU_max_. RU is the response units at the steady state level, C is the concentration of protein in the flow solution, *K_a_* is the association constant, RU_max_ is the maximum response units at saturation, and n is the number of binding sites on RNA. RU_max_ can be predicted using the following equation: RU_max_ = k*n*RU^R^*MW^P^/MW^R^, where RU^R^ is the amount of immobilized RNA in response units, MW^P^ and MW^R^ are molecular weights of protein and RNA respectively, and k is the refractive index increment ratio of RNA to protein [1]. As one SNF RRM1 only binds one U1 RNA, we were able to calculate the binding stoichiometry between SNF and poly(U) RNA by assuming n = 1 for SNF RRM1 binding U1 snRNA.

***Analytical ultracentrifugation***

The Sedimentation velocity experiments were carried out with a Beckman Coulter ProteomeLab^TM^ XL-I instrument. All AUC runs were carried out at the rotation speed of 60,000 rpm at 16°C. The sample volume was 400 μl and the protein concentration was about 1.2 mg/ml for SNF RRM1 and 2.2 mg/ml for SNF (^2^H, ^15^N, and ^13^C labeled sample) in 50 mM PBS buffer (pH 7.0, 1 mM EDTA). A wavelength of 280 nm was used to record the UV absorption of the cells which scanned every 30s for 5 h. Then the collected data were analyzed with the SedFit98 program.

Reference

1. Wilson WD, Wang L, Tanious F, Kumar A, Boykin DW, Carrasco1 C, Bailly C (2001) Biacore’s SPR technology and DNA footprinting for the discovery and development of new DNA-targeted therapeutics and reagents. Biacore J 1:15-20.
