# Supplementary figures and images for: Structure and Novel Functional Mechanism of Drosophila SNF in Sex-Lethal Splicing
Source: PLoS One. 2009 Sep 3;4(9):e6890. doi: 10.1371/journal.pone.0006890 (PMC2731243; doi:10.1371/journal.pone.0006890)

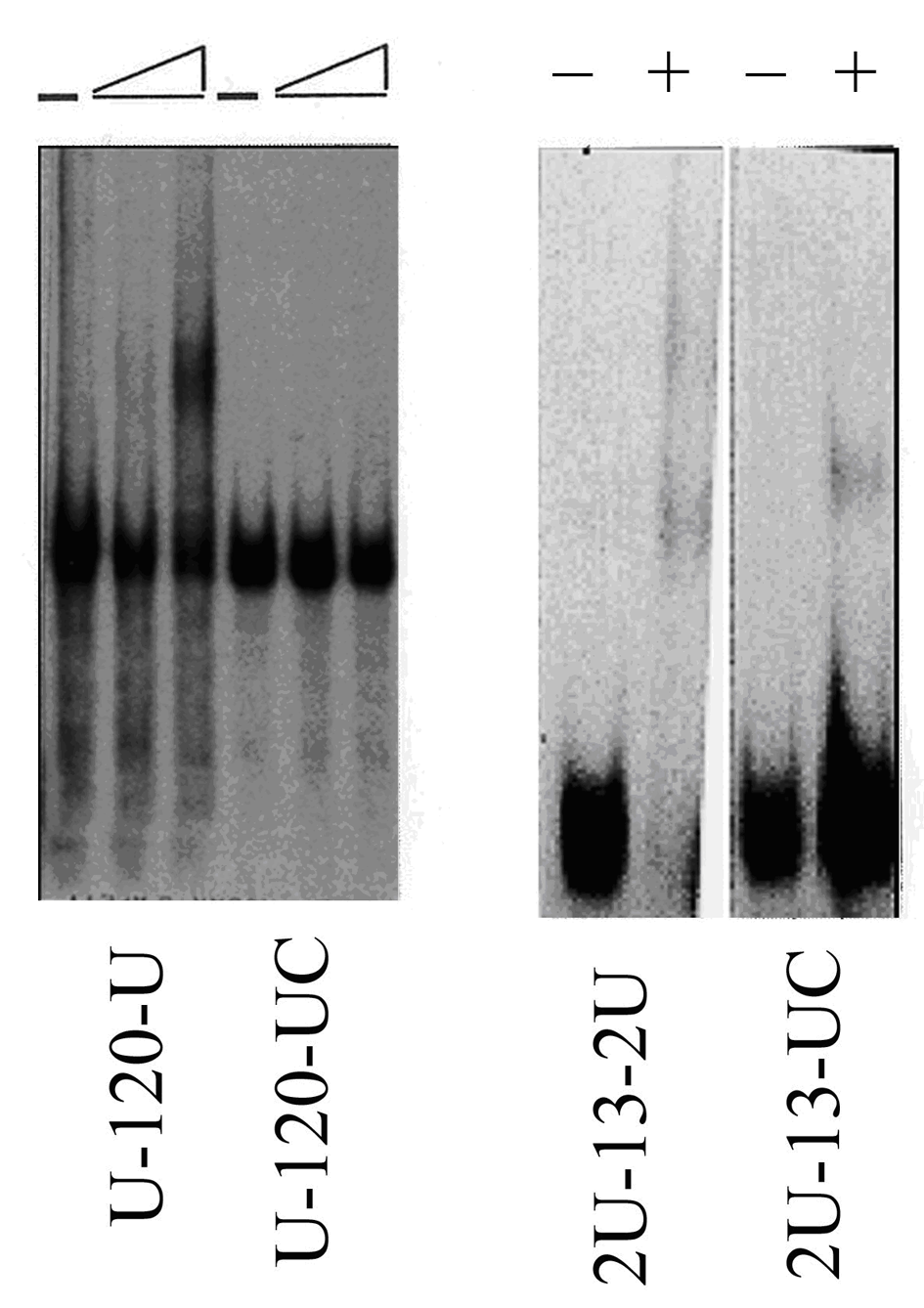

Supplement: Figure S1 — RNA binding assay for SNF and U-120-U (with two U-runs separated by 120 bases), U-120-UC (with one U-run), 2U-13-2U (with two double-U-runs separated by 13 bases) and 2U-13-UC (with one double-U-run) RNA substrates. (0.63 MB TIF) [file pone.0006890.s002.tif]

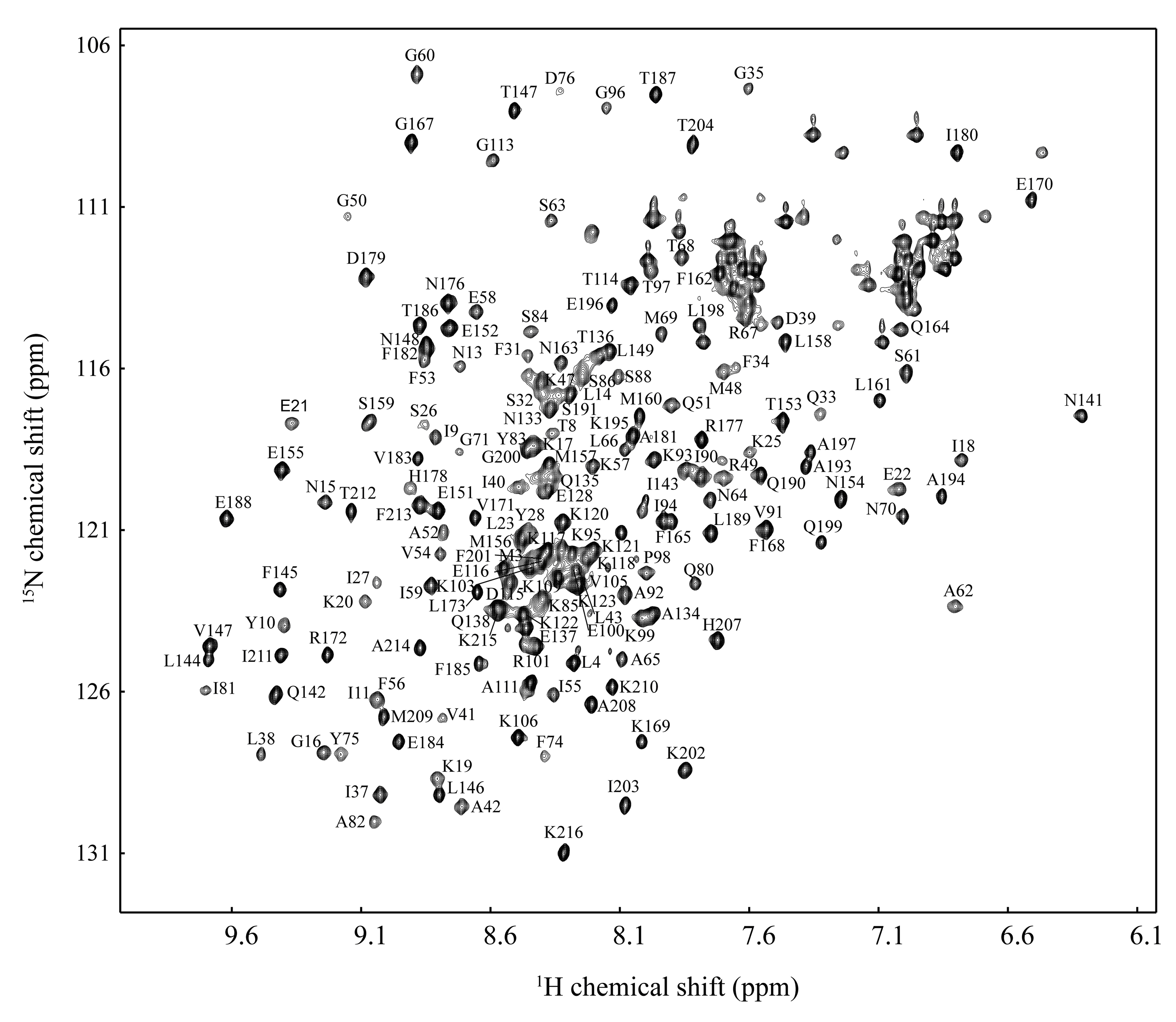

Supplement: Figure S2 — The 2D 1H-15N HSQC spectrum of full-length SNF at pH 7.2. Assignments are labeled. (1.41 MB TIF) [file pone.0006890.s003.tif]

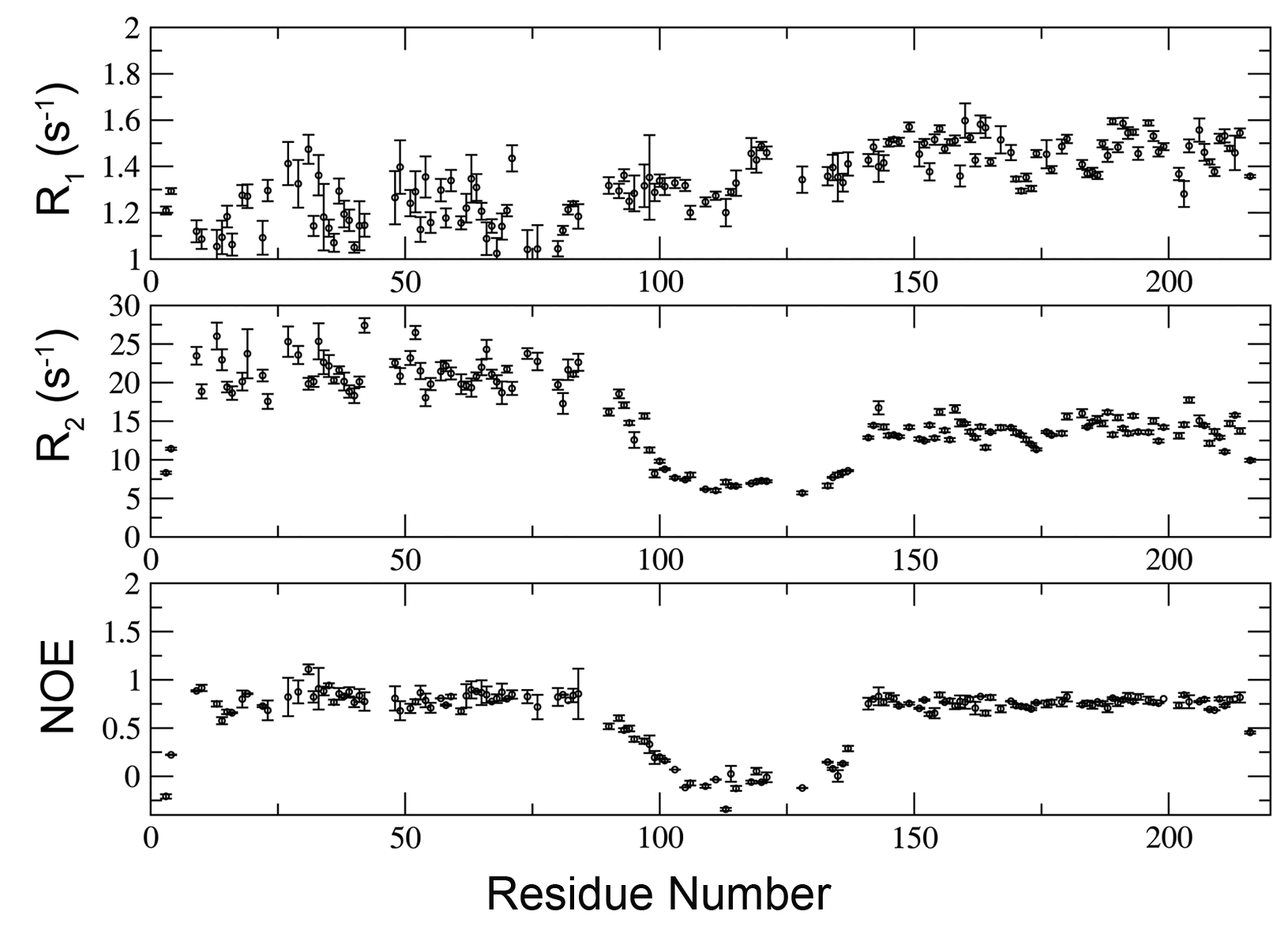

Supplement: Figure S3 — Backbone dynamics of full-length SNF. R1, R2, and heteronuclear {1H}-15N NOE values are plotted against residue numbers. (0.28 MB TIF) [file pone.0006890.s004.tif]

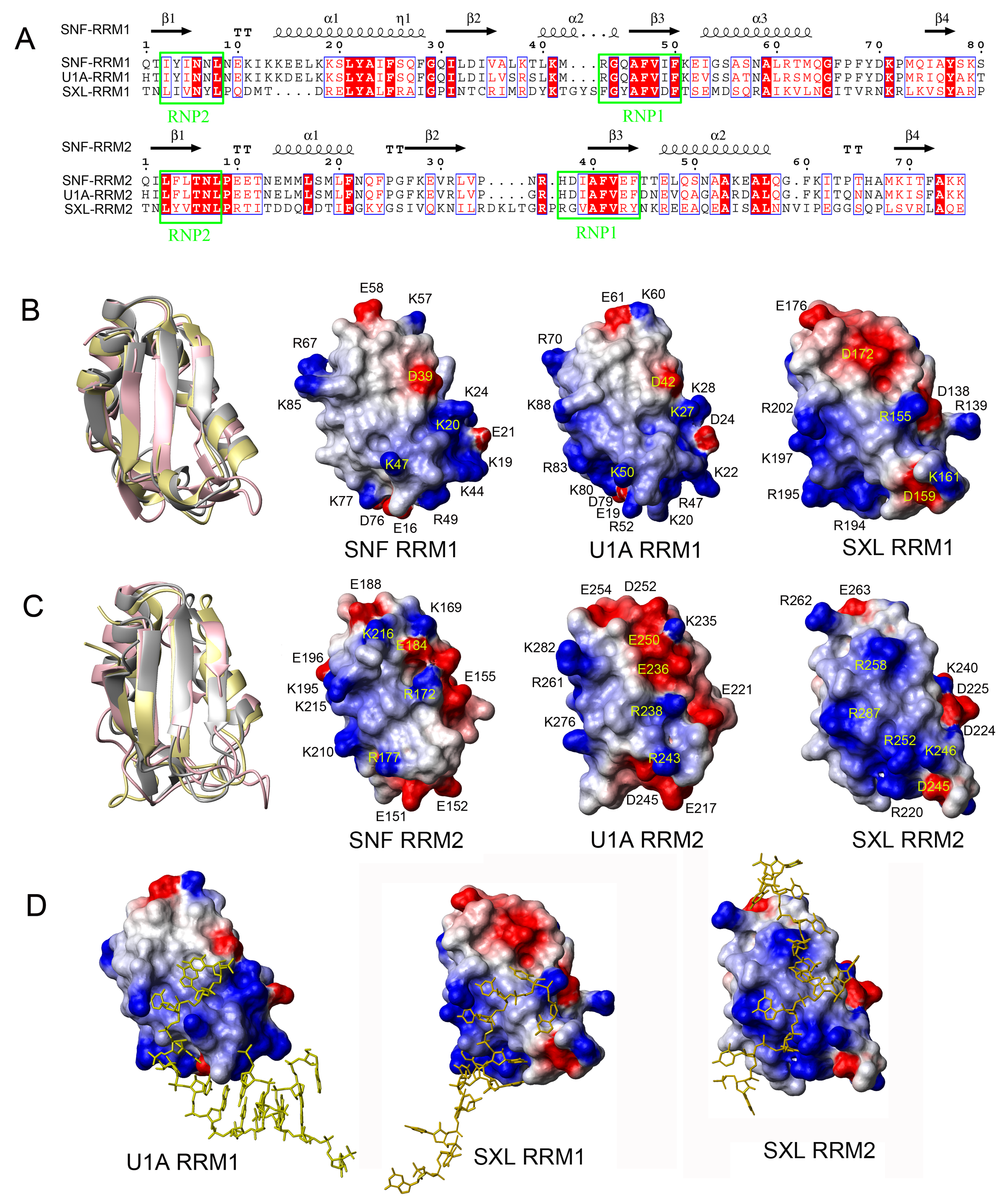

Supplement: Figure S4 — Sequence alignment and surface charge comparison. (A) Structure based sequence alignment of RRM1 and RRM2 in SNF (residues 7–86 and residues 142–216), U1A (residues 10–90 and residues 207–282) and SXL (residues 125–203 and residues 211–291). Conserved residues are shown in red. The secondary structure is displayed at the top. Residues involved in forming RNP1 and RNP2 are highlighted in green. (B) Comparison of the surface charge of SNF RRM1, U1A RRM1 and SXL RRM1. (C) Comparison of the surface charge of SNF RRM2, U1A RRM2 and SXL RRM2. The alignment of the three structures is shown on the left (SNF: grey; U1A: khaki; SXL: pink). Surface charge distribution of SNF RRM1, U1A RRM1 and SXL RRM1 are shown from left to right. (D) Surface charge distribution of U1A/U1hpII RNA complex (left) and SXL/GUUGUUUUUUUU complex (RRM1 shown in the middle, RRM2 shown on the right). Negatively charged residues are shown in red, and positively charged residues are shown in blue. (3.87 MB TIF) [file pone.0006890.s005.tif]

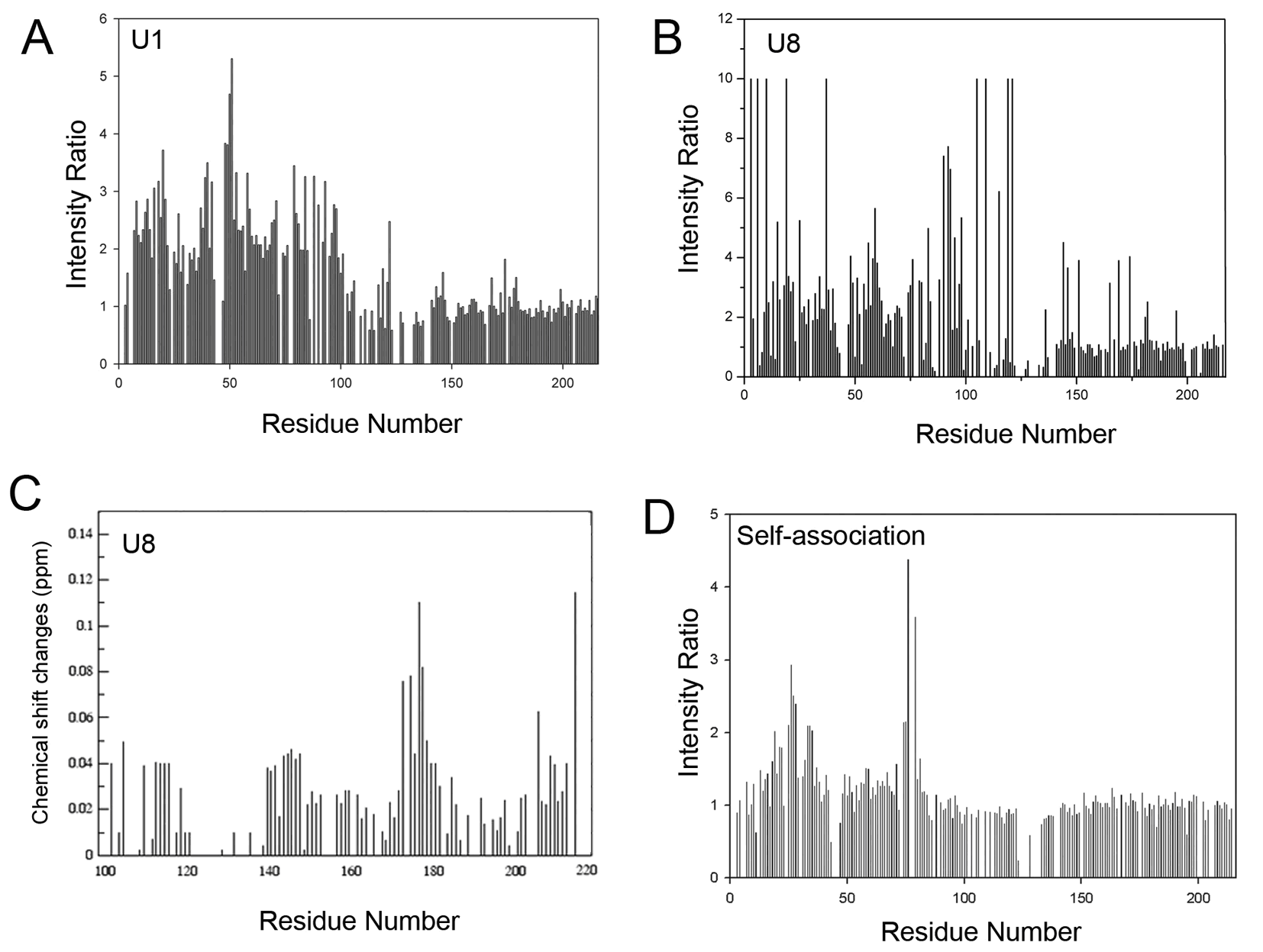

Supplement: Figure S5 — Bar plots displaying SNF residues that change in peak intensity or chemical shift on titration with U1hpII RNA,poly(U) RNA and self-association. (A) NH signal intensity ratio between free SNF and SNF/U1hpII (1∶7). The residues that display significant concentration-dependent NH peak intensity changes include I18, K20, D39, I40, A42, M48, R49, G50, Q51, F53 E58, M79, S84, S88 and K93 (Ifree/IU1>3). (B) NH signal intensity ratio between free SNF and SNF/poly(U) RNA (1∶15). The cut off is set to 10. The residues that display significant concentration-dependent NH peak intensity changes include M3, Y10, N15, K19, K25, I37, Y83, I90, V91, A92, K93, F98, V105, K109, D115, K121 and K122 (Ifree/IU8>5). (C) Changes in average NH chemical shifts of SNF RRM2 (plus the linker loop) on titration with poly(U) RNA (1∶15). The following residues exhibit obvious NH chemical shift changes (Δδcomb>0.04 ppm): K103, K106, T114, D115, E116, K117, F145, L146, T147, N148, K149, V174, N176-F182, H207, K210, I211, A214 and K216. (D) NH signal intensity ratio of SNF at 0.035 mM and 0.39 mM. The residues that display significant concentration-dependent NH peak intensity changes include K25, S26, L27, Y28, Q33, F34, G35, F74, Y75, D76 and M79 (I0.035 mM/I0.39 mM>3). (0.61 MB TIF) [file pone.0006890.s006.tif]

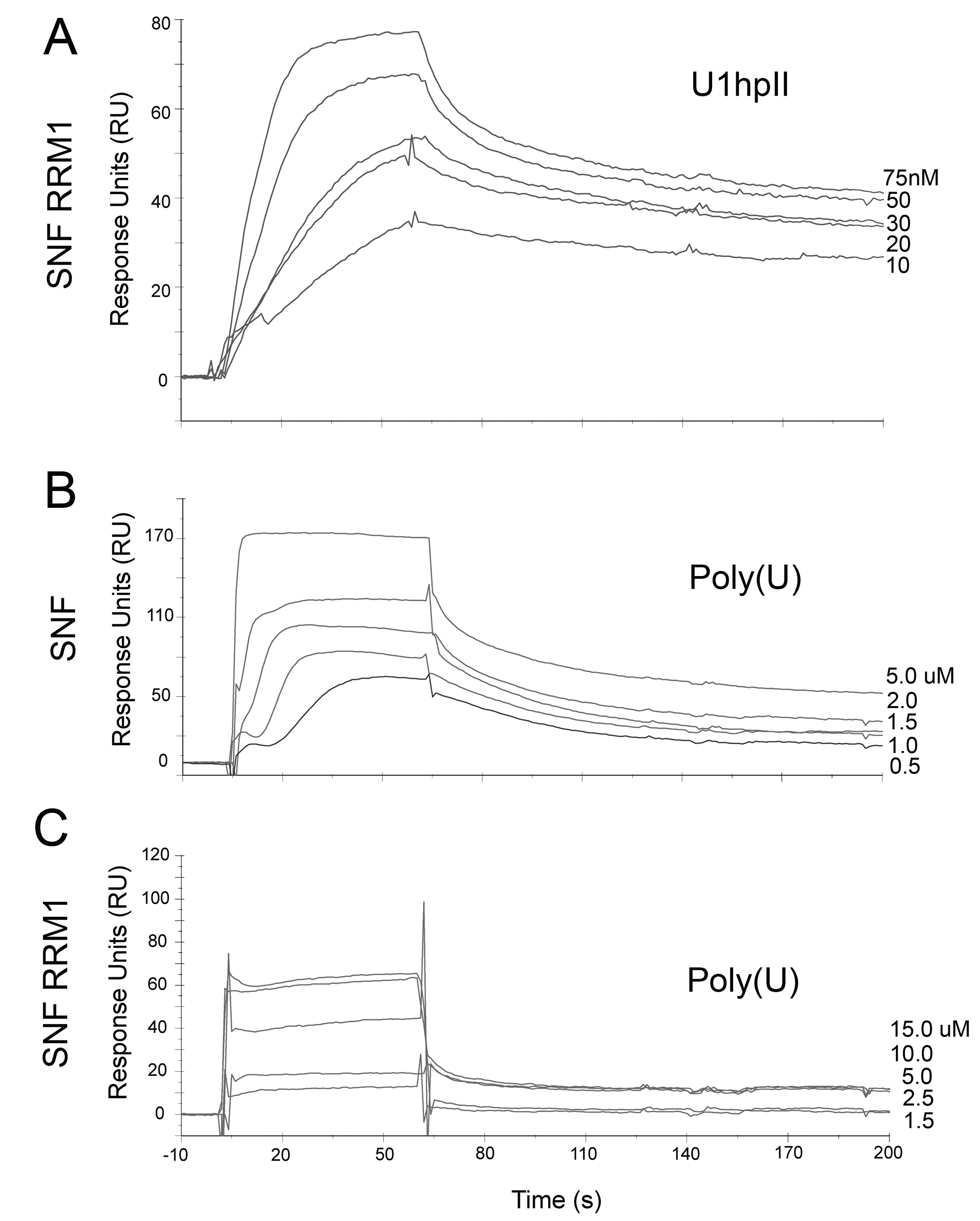

Supplement: Figure S6 — SPR analysis of the interactions between SNF (or SNF RRM1) with U1hpII RNA and poly(U) RNA. SPR analysis was carried out using BIACORE 3000 (Biacore), as described in Materials and Methods. Interactions between U1hpII RNA and SNF RRM1, poly(U) RNA and SNF, poly(U) RNA and SNF RRM1 are shown in A, B and C, respectively. Five different concentrations of protein injected over the RNA surfaces are shown in the right side. Scatchard-plot analysis of the protein-RNA interactions were carried using results from the above sensorgrams, and it is found that the number of binding sites (n) on poly(U) RNA is 1.06 for SNF and 1.20 for SNF RRM1, which are all close to 1∶1 binding stoichiometry. (0.82 MB TIF) [file pone.0006890.s007.tif]

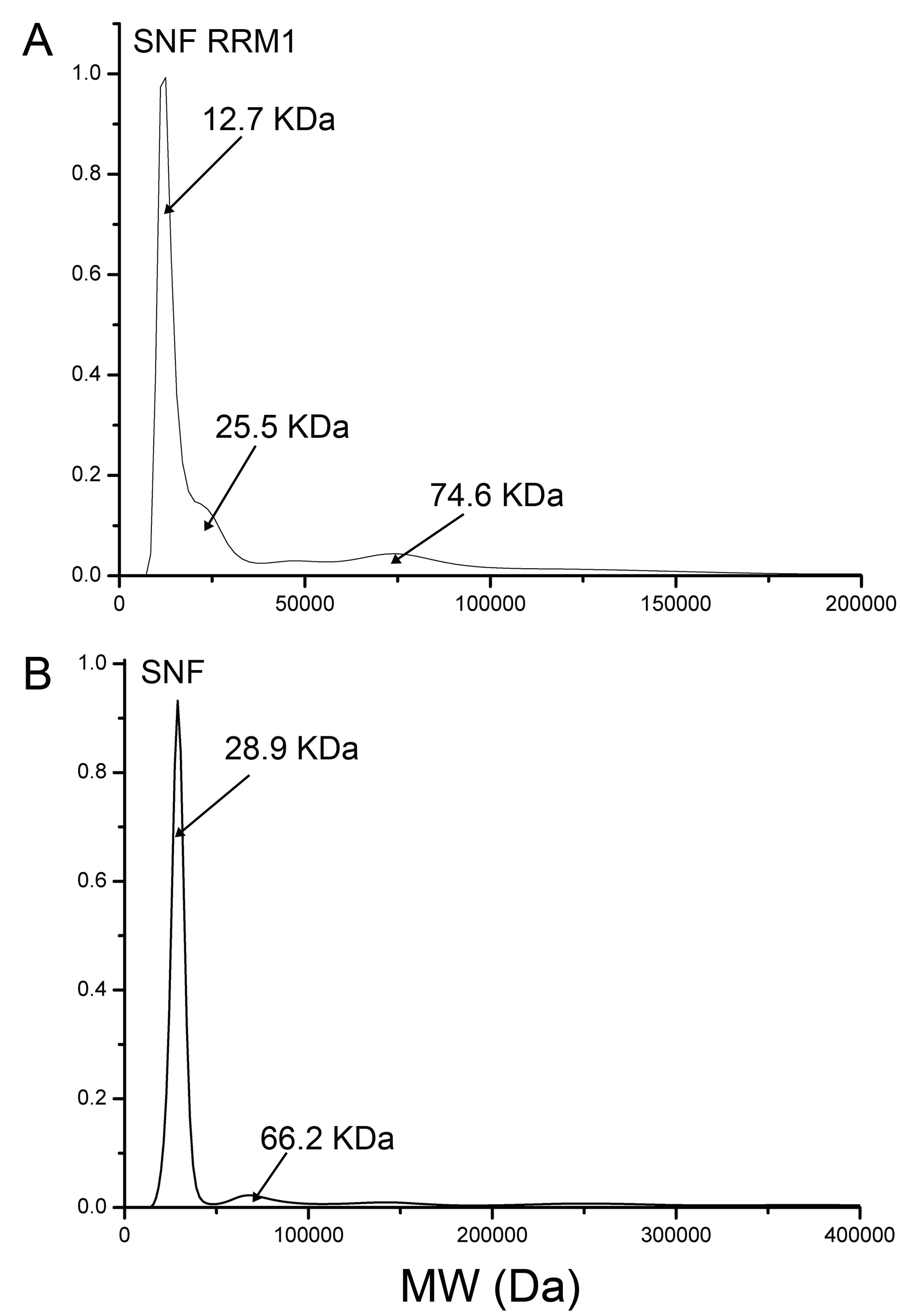

Supplement: Figure S7 — Analytical ultracentrifugation analysis of SNF RRM1 (A) and SNF (B). The protein concentration was about 0.1 mM for SNF RRM1 and 0.09 mM for SNF (2H, 15N, and 13C triple labeled sample), respectively. (0.32 MB TIF) [file pone.0006890.s008.tif]
